# Supplementary material for: Cervical cancer risk and access: Utilizing three statistical tools to assess Haitian women in South Florida
Source: PLoS One. 2021 Jul 6;16(7):e0254089. doi: 10.1371/journal.pone.0254089 (PMC8259954; doi:10.1371/journal.pone.0254089)
Supplement: Attachment — Submitted filename: PLOS_Rresponse to Reviewers_2021March01.docx [file pone.0254089.s001.docx]

**1.  In the Methods section of the manuscript, please ensure you have included the registration number for the clinical trial referenced in the manuscript. Furthermore, in the Methods section, please ensure that you have included the full names of the ethics committee/institutional review board(s) that approved the clinical trials referenced in the manuscript. Thank you for your attention to our requests.**

We thank you for bringing this to our attention and have updated the manuscript.

*“Data were extracted and merged from two larger studies, namely Health in Your Hands* ***(HIYA; Clinical Trial Registration Number NCT02970136)*** *and South Florida Center for the Reduction of Cancer Health Disparities* ***(SUCCESS; Clinical Trial Registration Number NCT02121548).*** *HIYA and SUCCESS tested modality, the fashion in which participants were screened (i.e., mailed self-sampler, community health worker [CHW] support). Both studies were ethically approved prior to the study by the* ***University of Miami ethics committee*** *as well as with an updated* ***University of Miami IRB*** *approval to be merged and analyzed (IRB #20180129).”*

**2.  Please kindly amend the blue text in your manuscript to black (Abstract).**

We thank you for bringing this to our attention and have updated the abstract.
