# Supplementary material for: Cervical cancer risk and access: Utilizing three statistical tools to assess Haitian women in South Florida
Source: PLoS One. 2021 Jul 6;16(7):e0254089. doi: 10.1371/journal.pone.0254089 (PMC8259954; doi:10.1371/journal.pone.0254089)
Supplement: Attachment — Submitted filename: PLOS_Rresponse to Reviewers_2021MAY18.docx [file pone.0254089.s002.docx]

**Reviewer #1: This study examined factors associated with cervical cancer screening among Haitian women. Specifically, the authors used three different statistical methods to determine salient factors related to lack of screening among. I have offered a few suggestions for the authors’ consideration:**

**Abstract**

1. **The authors state, “This study seeks to assess cancer risk and access of unscreened Haitian women.” The statement seems to indicate an examination of general cancer risk as opposed to risk and access specific to cervical cancer.**

We agree and included the word cervical to specify the cancer type.

**Introduction**

2. **The first sentence of the second paragraph is missing a closed parenthesis. Same issue for the last sentence of paragraph three.**

We agree and have updated the manuscript to have the missing closed parenthesis.

3. **The description of the influence of how structural violence influence health behaviors is an interesting concept. However, since the study focuses on specific determinants of cancer screening, which have been examined in previous immigrant health literature, it would be helpful to provide further justification for choosing the specific screening determinants in this study.**

We agree that the introduction would benefit from further justification. We previously limited this section for sake of word count; however, we now updated the manuscript to detail the reasoning for selecting specific screening determinants as well as citations for the structural violence influence on health behaviors.

4. **The authors indicate that the study utilizes three distinct statistical methods to examine factors associated with Pap screening. It seems to me that the focus of the paper is on the use of the statistical methods, so it would be helpful to provide additional justification for using this approach.**

We agree and you have shared insight on how to resolve this issue in response to your comment # 8 below. We have updated the manuscript as you suggested. Thank you for the insight.

**Methods**

5. **The theoretical rationale seems a bit out of place in the methods sections. I suggest including this in the introduction section. Perhaps, it may bolster the arguments for examining factors associated with Pap screening using three statistical methods.**

We agree and have moved the theory into the introduction as suggested.

6. **Why were data from the two studies merged, I assume both data were very similar? What was the sample sizes for each data?**

We included data from two studies in order to maximize the sample size. In order to meet the word count, we did not previously detail the studies. Further, we included their citations for reference as desired. Nonetheless, the studies and samples were similar. We also conducted preliminary data analysis to ensure there were no significant differences in the data to further justify merging. The sample was fairly split across both datasets (*n*=148 SUCCESS; *n*=198 HIYA). We have included this detail in the manuscript for clarity as well. The manuscript includes the following:

*“Data were extracted and merged from two larger studies testing modality (Health in Your Hands (HIYA) and South Florida Center for the Reduction of Cancer Health Disparities (SUCCESS) studies), the fashion in which participants get screened. Both studies were ethically approved prior to the study as well with an updated IRB approval to be merged and analyzed (IRB #20180129). Both studies, HIYA and SUCCESS included purposive convenience sampling. SUCCESS sought to bypass barriers to screening through delivering HPV self-sampling tools to women via a community health worker (CHW). Indeed, the study produced significant results indicating efficacy in screening uptake despite women’s lack of knowledge, insurance, and access. HIYA sought to clarify the viability of HPV self-sampling tools delivered by mail in order to circumvent conflict or confusion with scheduling an appointment with a CHW. Findings from HIYA suggested no statistical differences in screening uptake by CHW compared to mailed kit, validating the feasibility of a new format for screening via a mailed self-sampling tool approach. Details of these studies may be found elsewhere (4, 34). We also conducted preliminary data analysis to ensure there were no significant differences in the data to further justify merging. The sample was fairly split across both datasets (n=148 SUCCESS; n=198 HIYA) to include n=346 Haitian women living in South Florida who were at risk for cervical cancer, defined as self-reported age of 30-65 and unscreened in the previous three years.”*

**Results**

7. **In subsection 3.2 (LRC, CART, and…) include the relevant statics, e.g. the ORs and CIs.**

We thank you for this feedback. We did not include this previously to maximize word count and readability. Please note, for instance, that there are multiple ORs and CIs for each LR as well as the two types of LR conducted (univariate and multivariate). This information is in the associated Tables and Figures. If this information is strongly suggested, despite the aforementioned concerns, we are happy to include it. Kindly, please let us know.

**Discussion**

8. **The first paragraph of the discussion provides more detailed rationale for utilizing the three statistical methods in this paper. I suggest incorporating some of the information here in the introduction section.**

We agree and have included the following language in the introduction:

*“Statistical approaches vary in their degree of responding to scientific inquiry due to intrinsic assumptions for each methodology; therefore, this study includes three different methods to curate a comprehensive view of Haitian women’s risk profiles for cervical cancer using predictors across immigration history, patterns of healthcare utilization, and key sociodemographic characteristics. Further, multiple statistical methodology allows for examination of similarities and nuances across analysis, producing more reliable results and more detailed findings.”*

**Tables**

9. **In table 1, I suggest including information for the categorical variables. For instance, what is the breakdown of length in the US, education, age etc.?**

We appreciate your feedback, but it is unclear what you suggest. There are two descriptive tables. Table 1 includes categorical variables. Table 2 includes continuous variables. Length in the US and age are continuous and the average and range are included. Education is binary (high school educated and above or less than high school educated). Please advise how we can improve this for ease of reference.

**Reviewer #2:**

**Introduction:**

1. **Your problem specification needs to be clearer, for example in page 8, you mentioned “structural violence, colonialism, racism and xenophobia” which are note in your aim of study.**

We agree and have reorganized the introduction to better guide the reader in our study’s problem specification. We also have addressed this comment in response to Reviewer #1 Comment #3.

2. **You should specify the reference for each sentence in your introduction, for example in page 9 the sentence:” Colonialism, sexism, racism, and xenophobia create a dynamic interplay of poverty, gendered experiences, and race consciousness, which must be exposed and rectified in order to promote health and prevent disease for all with efficiency and sustainability.” Has not any references. There are many such sentences that you need to add references for them.**

We agree and have addressed this comment in response to Reviewer #1 Comment #3 and your previous comment #1.

3. **At the end of the introduction, please mention the aim of your study.**

We agree and have included the following language.

*“Overall, this study aims to understand the characteristics of a Haitian woman living in South Florida who is at-risk for cervical cancer defined by her self-reported health history including absence of cervical cancer screening with a Pap test. Statistical findings may help to provide better comprehension of sociocultural and socioenvironmental factors fostering (or impeding) women immigrants’ ability to navigate the U.S. healthcare system. Results may inform public health planning and delivery for Haitian women as well as other underserved immigrants living in the US for equitable access to disease prevention and control.”*

**Methods:**

4. **Please explain why you chose PEN 3-Cultural Model for your study.**

We agree the selection of the model needs to be made clearer. We have added this language earlier in the paper (introduction section now includes a theoretical rationale). We have also expanded the justification of the model. For instance, the manuscript includes the following:

*“Further, the PEN-3 Cultural Model has several domains which help researchers approach data analysis and interpretation with cultural sensitivity (29-32)”*

5. **In page 10, part 2.1 the last sentence should be transferred to the introduction.**

We agree and have moved the entire 2.1 section into the introduction in response to Reviewer #1’s comment #5.

6. **Please also mention the criteria and reason you considered for selecting each predictor.**

We agree and have addressed this comment in response to Reviewer #1 Comment #3.

7. **Since the number of your data is low, the results are not reliable, so please try applying cross validation to gain more reliable results.**

We appreciate this feedback and agree the sample size is relatively low for the type of analysis we conducted. However, we have also included data validation metrics in our analysis which demonstrates the reliability of the results through measures such as accuracy. We note this as a limitation of our study as well as an innovative element and strength. The following language is included in the manuscript:

*“Additionally, the sample size was relatively small for the type of analyses typically used for machine learning tools (50-54). The sample also included a skewed sample of Haitian women with low SES indicated by a majority of below poverty or unknown income. In spite of the limited, medium sample size, with low SES Haitian women, the consistency of results along with their associated data validation metrics producing accuracy across all methods are encouraging. “*

**Results:**

8. **The accuracy obtained from the approaches may be improved, try selecting more important variables for your prediction and report them if the results improved.**

We truly appreciate your feedback. In fact, we have conducted extensive data modeling and assessment prior to submitting. For instance, we developed a logistic regression model that was theory based as well as data driven. Further, the machine learning methods followed a similar approach. We ensured the best model was built based off of the important variables prior to submitting. The accuracy may be increased with a larger sample. We are considering this for future studies. Thank you.

**EDITORIAL COMMENTS**

**1.  In the Methods section of the manuscript, please ensure you have included the registration number for the clinical trial referenced in the manuscript. Furthermore, in the Methods section, please ensure that you have included the full names of the ethics committee/institutional review board(s) that approved the clinical trials referenced in the manuscript. Thank you for your attention to our requests.**

We thank you for bringing this to our attention and have updated the manuscript.

*“Data were extracted and merged from two larger studies, namely Health in Your Hands* ***(HIYA; Clinical Trial Registration Number NCT02970136)*** *and South Florida Center for the Reduction of Cancer Health Disparities* ***(SUCCESS; Clinical Trial Registration Number NCT02121548).*** *HIYA and SUCCESS tested modality, the fashion in which participants were screened (i.e., mailed self-sampler, community health worker [CHW] support). Both studies were ethically approved prior to the study by the* ***University of Miami ethics committee*** *as well as with an updated* ***University of Miami IRB*** *approval to be merged and analyzed (IRB #20180129).”*

**2.  Please kindly amend the blue text in your manuscript to black (Abstract).**

We thank you for bringing this to our attention and have updated the abstract.
